# Homocysteine concentration in coronary artery disease and severity of coronary lesions

**DOI:** 10.1111/jcmm.18474

**Published:** 2024-06-19

**Authors:** Zhi Luo, Kai Tang, Gang Huang, Xiao Wang, Shiheng Zhou, Daying Dai, Hanxuan Yang, Wencai Jiang

**Affiliations:** ^1^ Department of Cardiology Suining Central Hospital Suining Sichuan China

**Keywords:** 5,10‐methylenetetrahydrofolate reductase, coronary artery disease, homocysteine, multiple vessel lesions

## Abstract

Our previous study reckons that the impact of the rs1801133 variant of 5,10‐methylenetetrahydrofolate reductase (MTHFR) on coronary artery disease (CAD) is possibly mediated by cardiometabolic disorder. This study is performed to verify this hypothesis. Four hundred and thirty CAD patients and 216 CAD‐free individuals were enrolled in this case–control study. The rs1801133 variant was genotyped by PCR‐RFLP. Severity of coronary lesions was evaluated by number of stenotic coronary vessels and extent of coronary stenosis. The rs1801133 T allele significantly increased homocysteine levels in patients with CAD and CAD‐free individuals. Individuals with the T allele of rs1801133 had an increased risk of developing CAD. In contrast, individuals with the TT genotype of rs1801133 were at high risk of multiple vessel lesions. The carriers of CT genotype had higher levels of systolic blood pressure (SBP), low‐density lipoprotein cholesterol (LDL‐C), and high‐sensitivity C‐reactive protein (hs‐CRP), and lower levels of apolipoprotein A1 (APOA1) than those with CC genotype in male patients with CAD. The receiver operating characteristic (ROC) curve and precision‐recall (PR) curve indicated that hyperhomocysteinemia was sensitive to predict the severity of CAD. Multivariate logistic regression revealed that homocysteine, rs1801133, age, smoking, weight, body mass index (BMI), lipoprotein(a) [Lp(a)], and hs‐CRP were independent risk factors for CAD. The increased risk of CAD and severity of coronary lesions associated with rs1801133 in the Chinese Han population were attributed, at least partly, to high homocysteine levels. Hyperhomocysteinemia had a high predictive value for severe CAD or multiple vessel lesions.

## 1 INTRODUCTION

The 5,10‐methylenetetrahydrofolate reductase (MTHFR) is a critical rate‐limiting enzyme in one‐carbon metabolism, which plays a key role in catalysing the conversion of 5,10‐Methylenetetrahydrofolate (5,10‐MTHF) to 5‐Methyltetrahydrofolate (5‐MTHF). 5‐MTHF is a direct one‐carbon donor (methyl group) for many substrates such as homocysteine,^1^ DNA,^2^ RNA,^3^ and proteins.^4^

The rs1801133 variant (also known as the 677C > T variant) is located at exon 4 and formed by a transition from cytosine (C) to thymine (T). The 222nd genetic code of the MTHFR gene is changed accordingly from GCC to GTC, resulting in the replacement of alanine (Ala) by valine (Val) in the MTHFR polypeptide. The C and T alleles encode high and low activity of MTHFR.^5^ Individuals with the TT genotype are predisposed to mild to moderate hyperhomocysteinemia.^6^, ^7^, ^8^

Plasma homocysteine levels are linked to cardiometabolic parameters in the Chinese population. For instance, Shih et al.^9^ found that plasma homocysteine levels were related to high‐density lipoprotein cholesterol (HDL‐C), fasting plasma glucose (FPG), systolic blood pressure (SBP), diastolic blood pressure (DBP), uric acid (UA) and body mass index (BMI). In addition, Chen et al.^10^ found that plasma homocysteine levels were negatively associated with HDL‐C levels and positively correlated with low‐density lipoprotein cholesterol (LDL‐C) levels and [(total cholesterol (TC)‐HDL‐C)/HDL‐C]. In contrast, Zhou et al.^11^ found that plasma homocysteine levels were negatively associated with HDL‐C, apolipoprotein A1 (APOA1) and lipoprotein(a) [Lp(a)], but positively correlated with triglyceride (TG) after adjusting BMI, SBP, DBP, FPG, UA, alanine aminotransferase (ALT) and aspartate aminotransferase (AST). Whereas cardiometabolic risk factors, such as dyslipidemia,^12^, ^13^ dysglycemia,^13^, ^14^ and hypertension^13^, ^15^ are closely associated with coronary artery disease (CAD) and severity of coronary lesions,^16^, ^17^, ^18^ it indicates that high homocysteine levels or hyperhomocysteinemia may induce CAD and multiple vessel lesions by inducing cardiometabolic disorder. Since plasma homocysteine levels are primarily determined by variant of rs1801133,^19^, ^20^, ^21^ it is tempting to speculate that the rs1801133 variant may influence the risk of CAD and the severity of coronary lesions in the Chinese population.

In fact, some studies have already investigated the association between rs1801133 and CAD in the Chinese population. For instance, Yu et al.^22^ included 1142 patients with CAD and 1106 age‐ and gender‐matched controls and found that rs1801133 T allele increased the risk of CAD. In contrast, Bennett et al.^23^ included 156,000 individuals and did not detect a positive correlation between rs1801133 and CAD, consistent with a large‐scale clinical trial conducted by Lewis et al..^24^ Since the present results were controversial,^22^, ^23^, ^24^ Li et al.^25^ performed a meta‐analysis (6117 patients with CAD and 5984 controls) to clarify or confirm whether rs1801133 affected the risk of CAD in Chinese individuals in the light of evidence‐based medicine. Their results showed that rs1801133 T allele (odds ratios [OR] = 1.65, 95% confidence intervals [CI] = 1.43–1.89, *p* < 0.0001) and TT genotype (OR = 1.48, 95% CI = 1.29–1.70, *p* < 0.0001) significantly increased the risk of CAD in Chinese individuals.^25^ However, the specific mechanism is not clear. Here, this study is conducted to investigate the impacts of rs1801133 on cardiometabolic parameters, to provide some clues or references for the clarification of underlying mechanism involved.

Despite numerous studies indicating that hyperhomocysteinemia is an independent risk factor for multiple cardiovascular diseases (e.g. atherosclerosis, CAD and hypertension, etc.),^26^ the recognition of hyperhomocysteinemia as a cardiovascular risk factor has been challenged by some high‐quality randomized clinical trials (RCTs)^27^, ^28^, ^29^ and meta‐analysis.^30^, ^31^ For instance, Toole et al.^27^ conducted a double‐blind RCT (the Vitamin Intervention for Stroke Prevention) and found that lowering plasma homocysteine levels with high doses of folic acid, B6 and B12 did not prevent recurrent stroke, CAD and death after stroke in Caucasian population.^27^ Consistent with another RCT (the Heart Outcomes Prevention Evaluation) (2) whereby Lonn et al.^28^ demonstrated that inhibiting hyperhomocysteinemia with folic acid and B vitamins failed to reduce the risk of major cardiovascular events in Caucasian population. In addition, a recent RCT (the Norwegian Vitamin) conducted by Bønaa et al.^29^ revealed that reducing plasma homocysteine levels with folic acid and B vitamins did not lower the risk of recurrent cardiovascular disease after acute myocardial infarction (AMI) in Caucasian population. In line with these RCTs,^27^, ^28^, ^29^ the findings from two meta‐analysis^30^, ^31^ directly refuted the causal relevance of moderately elevated homocysteine concentrations and homocysteine‐related pathways for CAD in Caucasian population. Taken together, hyperhomocysteinemia seems to have no relationship with CAD in Caucasian individuals. Nevertheless, hyperhomocysteinemia appears to have a correlation with CAD in Chinese individuals.^32^ Here, this study is conducted to investigate whether hyperhomocysteinemia causally increase the risk of CAD and severity of coronary lesions in Chinese individuals via a comprehensive and multidimensional analysis.

## 2 METHODS

### 2.1 Study participants

**FIGURE 1:**
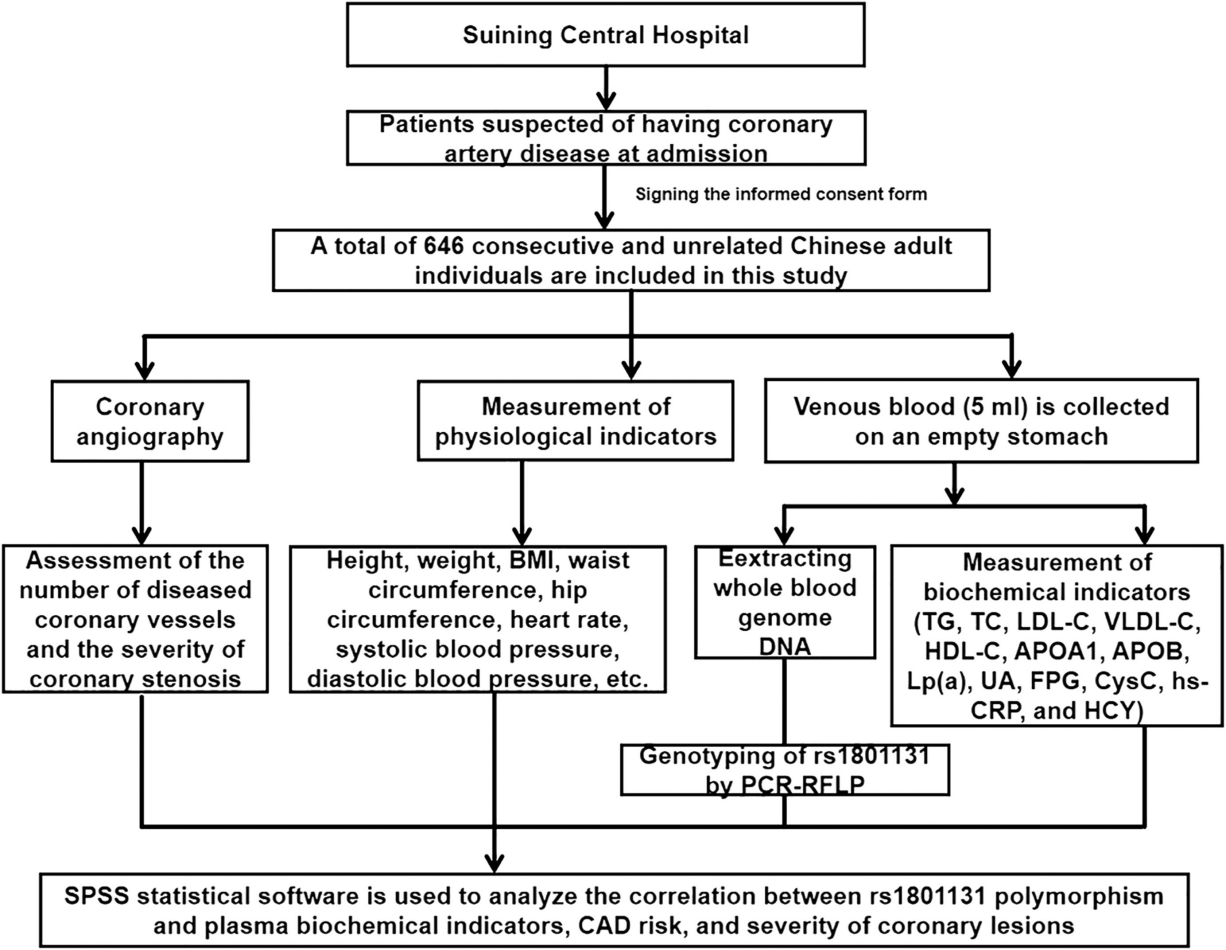
The flow chart of this research. ApoAI, apolipoprotein AI; ApoB, apolipoprotein B; BMI, body mass index; CAD, coronary artery disease; CysC, cystatin c; FPG, fasting plasma glucose; HCY: homocysteine; HDL‐C: high‐density lipoprotein cholesterol; hs‐CRP, hypersensitive C reactive protein; LDL‐C, low‐density lipoprotein cholesterol; Lp(a), lipoprotein (a); PCR‐RFLP, polymerase chain reaction‐restriction fragment length polymorphism;TC, total cholesterol; TG, triglycerides; UA, Uric acid; VLDL‐C, very low‐density lipoprotein cholesterol.

A total of 646 consecutive and unrelated Chinese adult individuals who underwent coronary angiography for suspected CAD at the Department of Cardiology, Suining Central Hospital were enrolled in the study (see Figure 1 for more details). Among these individuals, 430 patients were diagnosed with CAD, while the remaining 216 individuals were free of CAD and considered as controls. The exclusion criteria are as follows: (1) individuals who were taking the drugs that may affect the metabolic parameters, for example, lipid‐lowering drugs; (2) individuals who had undergone percutaneous coronary intervention; (3) individuals with active inflammatory disease, renal or hepatic disease, myocarditis or malignant disease. The research protocol was reviewed and approved by the Ethics Committee of Suining Central Hospital. All the participants signed an informed consent prior to participation in the study. The tenets of the Declaration of Helsinki were followed in all the research procedures described in this paper.

### 2.2 CAD diagnosis and severity evaluation

CAD was diagnosed if one or more major coronary arteries had stenosis greater than 50%. The individuals with coronary stenosis less than 50%, coronary atherosclerosis and healthy coronary arteries were defined as CAD‐free controls. Standard coronary angiography with multiple views at the right and left coronary arteries was performed by using Philips Medical Systems Nederland B.V. (Azurion 3 M15). The extent of coronary stenosis and the number of stenotic coronary vessels were used to assess the severity of CAD. CAD severity by the extent of coronary stenosis was defined as mild, moderate, severe, and very severe for 50%–74% stenosis, 75%–90% stenosis, 91%–99% stenosis and 100% stenosis, respectively, in any of the major arteries. CAD severity by number of stenotic coronary vessels was defined as the sum of the major coronary arteries with stenosis greater than 50%, including left main coronary artery (LM) stenosis (two branches), left anterior descending branch (LAD) stenosis (one branch), left circumflex branch (LCX) stenosis (one branch) and right coronary artery (RCA) stenosis (one branch).

### 2.3 Biochemical measurement

Overnight fasting blood samples were collected on the first morning after hospitalization of the individuals and blood samples were sent to Department of Clinical Laboratory for the measurement of TG, TC, LDL‐C, very low⁃density lipoprotein cholesterol (VLDL‐C), HDL‐C, APOA1, apolipoprotein B (APOB), Lp(a), UA, FPG, cystatin C (CysC), hs‐CRP and homocysteine. TG, TC, LDL‐C, VLDL‐C, HDL‐C, UA, FPG and homocysteine were measured by using the enzymatic method. APOB, APOA1, Lp(a), CysC and hs‐CRP were measured by using an immunoturbidimetric assay. An automatic clinical chemistry analyser (Beckman Coulter AU5800, USA) was used to measure all the parameters.

### 2.4 Genotyping

A 198‐bp fragment in the exon 4 of the MTHFR gene was amplified by using 40 pmol of each primer (forward: 5′‐TGAAGGAGAAGGTGTCTGGGGGA‐3′ and reverse: 5′‐AGGACGGTGCGGTGAGAGTG‐3′) (Sangon Biotech, China). PCR was performed in a volume of 20 μL containing 200 ng of genomic DNA, 10 μL of 2 × Taq MasterMix (CoWin Bioscience, Beijing, China), 6.25 μM (1.0 μL) of each primer, 5 μL ddH2O and 1 U of DNA polymerase (CoWin Bioscience, Beijing, China). The mixture was initially denatured at 95°C for 5 min, followed by 35 cycles of 95°C for 1 min, 59°C for 1 min and 72°C for 2 min, with a final 7 min extension at 72°C. The amplified PCR products were subjected to overnight restriction digestion by 5 U of HinfI (New England Biolabs, Beijing, China) at 37°C, and the restriction digestion products were separated on a 3% agarose gel and visualized by ethidium bromide staining. The mutant allele (677 T) gives HinfI restriction fragments of 175 bp and 23 bp, whereas the normal allele (C677), gives a single fragment of 198 bp.

### 2.5 Statistical analysis

Continuous variables between CAD and control groups were analysed by independent‐sample *t*‐test. Differences in the cardiometabolic parameters among the individuals with different genotypes in the CAD group or control group were analysed by one‐way ANOVA analysis for continuous variables. Differences in the frequencies of genotypes or alleles between CAD and control groups were analysed by *χ* ^2^ test. Differences in the frequencies of genotypes or alleles among the patients with different numbers of stenotic coronary arteries or extent of coronary stenosis were analysed by *χ* ^2^ test. *p* value less than 0.05 was considered statistically significant. Statistical analysis was performed using SPSS 25.0 (SPSS Inc., Chicago, IL, USA).

## 3 RESULTS

### 3.1 Clinical characteristics of the study population

The CAD group (*n* = 430) had older age (*p* < 0.001), and higher levels of SBP (*p* < 0.001), DBP (*p* < 0.001), TG (*p* = 0.05), LDL‐C (*p* = 0.01), APOB (*p* = 0.01), Lp(a) (*p* < 0.001), FPG (*p* < 0.01), CysC (*p* = 0.03), hs‐CRP (*p* < 0.001) and homocysteine (*p* < 0.001) than the control group (Table S1). The CAD group had lower levels of HDL‐C (*p* < 0.001) and APOA1 (*p* < 0.001) than the control group (Table S1). The variables including height (*p* = 0.73), weight (*p* = 0.21), BMI (*p* = 0.23), heart rate (HR) (*p* = 0.12), TC (*p* = 0.12), VLDL‐C (*p* = 0.44) and UA (*p* = 0.13) were comparable between the two groups (Table S1).

### 3.2 rs1801131 genotyping

Allele and genotype frequencies in both CAD and control groups are described in Table 1. The genotypes within CAD group (χ2 = 0.38, *p* = 0.54) and control group (*χ*2 = 1.19, *p* = 0.08) were distributed in accordance with the Hardy–Weinberg equilibrium (Table 1). rs1801131 T allele increased the risk of CAD in dominant model (OR = 1.70, 95% CI = 1.21–2.40, *p* < 0.01) (Table 1), but not in recessive model (OR = 1.75, 95% CI = 0.88–3.51, *p* = 0.11) (Table 1).

**TABLE 1:** Genetic characteristics of the study population.

	Control group	CAD group	*p* values	OR (95% CI)
*MTHFR* rs1801133 (All individuals)				
CC, *n* (%)	89 (41.0)	126 (29.0)	0.01	Dominant model (CT + TT vs. CC): 1.70 (1.21–2.40) Recessive model (TT vs. CC + CT): 1.75 (0.88–3.51)
CT, *n* (%)	116 (54.0)	267 (62.0)		
TT, *n* (%)	11 (5.0)	37 (9.0)		
χ2	1.19	0.38		
*P*_HWE_	0.08	0.54		
C allele, *n* (%)	294 (68.0)	519 (60.0)	0.01	
T allele, *n* (%)	138 (32.0)	341 (40.0)		
*MTHFR* rs1801133 (Male individuals)				
CC, *n* (%)	54 (45.3)	76 (27.8)	<0.01	Dominant model (CT + TT vs. CC): 2.15 (1.38–3.37) Recessive model (TT vs. CC + CT): 1.61 (0.68–3.84)
CT, *n* (%)	58 (48.7)	172 (63.0)		
TT, *n* (%)	7 (5.8)	25 (9.1)		
χ2	0.09	0.10		
*P*_HWE_	0.88	0.33		
C allele, *n* (%)	166 (69.7)	324 (59.3)	0.01	
T allele, *n* (%)	72 (30.2)	222 (40.6)		
*MTHFR* rs1801133 (Female individuals)				
CC, *n* (%)	35 (35.7)	50 (31.8)	0.47	Dominant model (CT + TT vs. CC): 1.19 (0.70–2.02) Recessive model (TT vs. CC + CT): 1.94 (0.61–6.21)
CT, *n* (%)	59 (60.2)	95 (60.5)		
TT, *n* (%)	4 (4.0)	12 (7.6)		
χ2	1.46	0.92		
*P*_HWE_	0.22	0.18		
C allele, *n* (%)	129 (65.8)	195 (62.1)	0.39	
T allele, *n* (%)	67 (34.1)	119 (37.8)		

Abbreviations: CAD, coronary artery disease; *MTHFR*, 5,10‐methylenetetrahydrofolate reductase; OR, odds ratio; 95% CI 95% confidence interval.

### 3.3 Impacts of rs1801131 on cardiometabolic parameters

The carriers of the TT genotype had higher homocysteine levels thsan the carriers of the CC genotype in patients with CAD (TT vs. CC = 16.40 ± 7.63 vs. 13.06 ± 4.46, *p* = 0.02) and CAD‐free (TT vs. CC = 16.78 ± 9.61 vs. 13.52 ± 4.51, *p* = 0.03) individuals (Table 2). In addition, the carriers of CT genotype had higher homocysteine (CT vs. CC = 14.95 ± 4.98 vs. 13.52 ± 5.12, *p* = 0.02), SBP (CT vs. CC = 148.29 ± 28.22 vs. 139.34 ± 24.65, *p* = 0.02), LDL‐C (CT vs. CC = 2.59 ± 0.99 vs. 2.32 ± 0.77, *p* = 0.04), and hs‐CRP (CT vs. CC = 15.14 ± 27.54 vs. 9.34 ± 17.84, *p* = 0.05) levels, and lower APOA1 levels (CT vs. CC = 0.98 ± 0.20 vs. 1.04 ± 0.15, *P* = 0.05) than those with CC genotype in male patients with CAD (Table 2). In contrast, the carriers of the TT genotype had higher LDL‐C levels (TT vs. CC = 2.88 ± 1.29 vs. 2.29 ± 0.60, *p* = 0.04) than those with CC genotype in male individuals without CAD (Table 2). Furthermore, the carriers of the TT genotype had higher homocysteine and FPG levels (TT vs. CC = 8.53 ± 5.81 vs. 6.30 ± 2.72, *p* = 0.03) than the carriers of the CC genotype in female patients with CAD (Table 2).

**TABLE 2:** Cardiometabolic characteristics of the individuals according to the *MTHFR* rs1801131 genotypes.

Control group					CAD group			*p* values
	CC genotype (*n* = 89)	CT genotype (*n* = 116)	TT genotype (*n* = 11)	*p* values	CC genotype (*n* = 126)	CT genotype (*n* = 267)	TT genotype (*n* = 37)	
Age, years	60.15 ± 10.22	62.16 ± 9.92	61.27 ± 9.56	0.36	64.86 ± 9.56	64.69 ± 9.33	62.76 ± 8.16	0.46
Height, cm	162.22 ± 7.06	161.46 ± 7.42	164.25 ± 7.63	0.50	160.48 ± 7.54	162.25 ± 7.37^a^	161.76 ± 7.70	0.03
Weight, kg	62.82 ± 10.42	63.52 ± 9.76	66.50 ± 9.05	0.53	60.80 ± 9.33	63.20 ± 9.67^a^	63.30 ± 9.57	0.02
BMI, kg/m^2^	23.58 ± 3.11	24.04 ± 3.30	24.17 ± 3.29	0.35	23.58 ± 3.11	24.04 ± 3.30	24.17 ± 3.29	0.38
HR, beats/minute	77.65 ± 14.90	75.68 ± 14.67	74.43 ± 13.12	0.27	77.65 ± 14.90	75.68 ± 14.67	74.43 ± 13.12	0.35
SBP, mmHg	137.38 ± 26.87	136.24 ± 22.78	131.00 ± 19.48	0.71	143.88 ± 26.94	147.86 ± 28.32	137.57 ± 24.15	0.07
DBP, mmHg	84.55 ± 15.71	83.03 ± 13.85	83.82 ± 13.10	0.76	89.40 ± 14.89	90.18 ± 16.72	85.76 ± 14.27	0.25
TG, mmol/L	1.35 ± 0.78	1.50 ± 0.84	1.35 ± 0.97	0.40	1.60 ± 1.26	1.59 ± 1.29	1.75 ± 0.95	0.75
TC, mmol/L	3.96 ± 0.93	4.13 ± 0.96	4.11 ± 1.33	0.47	4.18 ± 1.19	4.22 ± 1.16	4.02 ± 1.22	0.61
LDL‐C, mmol/L	2.32 ± 0.66	2.45 ± 0.69	2.60 ± 1.09	0.25	2.53 ± 0.90	2.64 ± 0.94	2.43 ± 0.96	0.32
VLDL‐C, mmol/L	0.56 ± 0.38	0.57 ± 0.30	0.44 ± 0.18	0.44	0.62 ± 0.47	0.58 ± 0.40	0.61 ± 0.30	0.68
HDL‐C, mmol/L	1.09 ± 0.25	1.10 ± 0.33	1.06 ± 0.27	0.88	1.03 ± 0.25	1.01 ± 0.26	0.97 ± 0.26	0.49
ApoAI, g/L	1.13 ± 0.16	1.13 ± 0.18	1.07 ± 0.20	0.64	1.08 ± 0.17	1.03 ± 0.21^a^	1.08 ± 0.18	0.05
ApoB, g/L	0.70 ± 0.23	0.76 ± 0.21	0.74 ± 0.32	0.19	0.78 ± 0.24	0.81 ± 0.28	0.73 ± 0.24	0.21
Lp(a), mg/L	199.13 ± 213.44	214.53 ± 236.46	236.21 ± 217.57	0.82	332.28 ± 384.03	297.91 ± 313.32	400.62 ± 389.96	0.20
UA, μmol/L	350.23 ± 116.57	347.40 ± 110.02	319.27 ± 93.49	0.71	364.37 ± 130.34	361.25 ± 109.23	363.28 ± 128.61	0.96
FPG, mmol/L	5.74 ± 1.47	6.17 ± 3.07	6.98 ± 0.65	0.22	6.63 ± 3.19	6.72 ± 3.08	6.93 ± 3.90	0.88
CysC, mg/L	0.75 ± 0.20	0.75 ± 0.21	0.74 ± 0.20	0.94	0.79 ± 0.29	0.85 ± 0.61	0.83 ± 0.28	0.62
hs‐CRP, mg/L	5.17 ± 12.12	3.73 ± 6.02	9.47 ± 19.22	0.15	8.74 ± 17.94	12.66 ± 24.69	4.92 ± 8.22	0.06
Homocysteine, μmol/L	13.52 ± 4.51	13.74 ± 4.63	16.78 ± 9.61^a^, ^b^	0.03	13.06 ± 4.46	14.03 ± 4.58^a^	16.40 ± 7.63^a^, ^b^	0.02

Male individuals without CAD					Male patients with CAD			
	CC genotype (*n* = 54)	CT genotype (*n* = 58)	TT genotype (*n* = 7)	*p* values	CC genotype (*n* = 76)	CT genotype (*n* = 172)	TT genotype (*n* = 25)	*p* values
Age, years	59.15 ± 10.99	63.21 ± 10.78^a^	60.57 ± 10.43	0.05	64.13 ± 10.11	64.72 ± 9.71	62.72 ± 8.78	0.61
Height, cm	166.40 ± 5.27	166.77 ± 5.99	167.20 ± 7.73	0.92	164.46 ± 6.38	166.02 ± 5.67	164.60 ± 7.25	0.14
Weight, kg	65.43 ± 9.81	67.20 ± 9.89	66.83 ± 8.70	0.64	63.77 ± 8.55	66.37 ± 8.26^a^	64.32 ± 9.78	0.03
BMI, kg/m^2^	23.61 ± 2.96	24.26 ± 3.41	24.55 ± 2.96	0.53	23.60 ± 2.99	24.12 ± 2.91	23.68 ± 3.01	0.41
HR, beats/minute	73.61 ± 13.96	73.38 ± 16.18	87.14 ± 20.54^ab^	0.03	78.39 ± 16.09	75.13 ± 15.49	73.32 ± 13.48	0.22
SBP, mmHg	141.24 ± 28.92	134.00 ± 23.22	128.71 ± 22.91	0.24	139.34 ± 24.65	148.29 ± 28.22^a^	138.96 ± 24.75	0.02
DBP, mmHg	86.22 ± 16.91	81.24 ± 13.40	84.14 ± 12.70	0.23	88.07 ± 14.38	90.70 ± 16.95	86.24 ± 14.47	0.27
TG, mmol/L	1.37 ± 0.90	1.47 ± 0.93	1.25 ± 0.73	0.75	1.46 ± 1.15	1.52 ± 1.33	1.58 ± 0.63	0.91
TC, mmol/L	3.93 ± 0.86	3.96 ± 0.98	4.46 ± 1.48	0.38	3.90 ± 0.97	4.12 ± 1.26	4.01 ± 1.24	0.41
LDL‐C, mmol/L	2.29 ± 0.60	2.43 ± 0.71	2.88 ± 1.29^b^	0.04	2.32 ± 0.77	2.59 ± 0.99^a^	2.43 ± 1.00	0.04
VLDL‐C, mmol/L	0.55 ± 0.39	0.52 ± 0.52	0.50 ± 0.16	0.87	0.58 ± 0.51	0.55 ± 0.33	0.63 ± 0.33	0.15
HDL‐C, mmol/L	1.08 ± 0.25	1.03 ± 0.38	1.07 ± 0.30	0.68	0.98 ± 0.22	0.96 ± 0.25	0.95 ± 0.25	0.68
ApoAI, g/L	1.13 ± 0.16	1.09 ± 0.20	1.07 ± 0.23	0.42	1.04 ± 0.15	0.98 ± 0.20^a^	1.05 ± 0.15	0.05
ApoB, g/L	0.69 ± 0.22	0.76 ± 0.22	0.83 ± 0.37	0.16	0.75 ± 0.22	0.79 ± 0.29	0.75 ± 0.26	0.46
Lp(a), mg/L	211.40 ± 249.93	198.27 ± 209.62	198.72 ± 122.60	0.95	327.36 ± 390.90	285.12 ± 298.39	390.74 ± 360.07	0.27
UA, μmol/L	367.55 ± 109.44	389.17 ± 105.74	343.12 ± 97.83	0.40	379.49 ± 130.34	385.72 ± 109.77	392.34 ± 132.51	0.88
FPG, mmol/L	5.73 ± 1.52	6.52 ± 3.80	4.93 ± 0.71	0.21	6.84 ± 3.47	6.71 ± 3.15	6.23 ± 2.52	0.71
CysC, mg/L	0.77 ± 0.20	0.78 ± 0.24	0.72 ± 0.13	0.79	0.81 ± 0.32	0.90 ± 0.73	0.86 ± 0.32	0.68
hs‐CRP, mg/L	5.66 ± 14.56	5.12 ± 7.95	13.57 ± 23.68	0.24	9.34 ± 17.84	15.14 ± 27.54^a^	5.47 ± 9.54	0.05
Homocysteine, μmol/L	14.59 ± 4.91	14.98 ± 4.12	18.12 ± 11.98	0.24	13.52 ± 5.12	14.95 ± 4.98^a^	15.54 ± 8.53	0.02

Female individuals without CAD					Female patients with CAD			
	CC genotype (*n* = 35)	CT genotype (*n* = 58)	TT genotype (*n* = 4)	*p* values	CC genotype (*n* = 50)	CT genotype (*n* = 95)	TT genotype (*n* = 12)	*p* values
Age, years	61.69 ± 8.83	61.10 ± 8.94	62.50 ± 9.14	0.92	65.96 ± 8.62	64.63 ± 8.65	62.83 ± 7.06	0.46
Height, cm	155.82 ± 3.99	156.35 ± 4.51	159.33 ± 5.13	0.39	154.58 ± 4.74	155.64 ± 4.95	155.83 ± 4.80	0.44
Weight, kg	58.86 ± 10.18	59.97 ± 8.27	66.00 ± 10.89	0.32	56.34 ± 8.73	57.81 ± 9.54	61.17 ± 9.13	0.25
BMI, kg/m^2^	24.15 ± 3.95	24.54 ± 3.02	26.00 ± 3.86	0.63	23.54 ± 3.30	23.89 ± 3.88	25.19 ± 3.74	0.38
HR, beats/minute	72.46 ± 13.20	75.00 ± 12.00	68.75 ± 10.43	0.44	76.52 ± 12.94	76.65 ± 13.13	76.75 ± 12.57	0.99
SBP, mmHg	131.43 ± 22.46	138.33 ± 22.36	135.00 ± 13.51	0.35	150.78 ± 28.99	147.11 ± 28.62	134.67 ± 23.63	0.21
DBP, mmHg	81.97 ± 13.46	84.69 ± 14.17	83.25 ± 15.77	0.66	91.44 ± 15.55	89.24 ± 16.33	84.75 ± 14.42	0.40
TG, mmol/L	1.31 ± 0.57	1.53 ± 0.74	1.52 ± 1.42	0.37	1.79 ± 1.38	1.71 ± 1.20	2.11 ± 1.38	0.59
TC, mmol/L	4.01 ± 1.04	4.29 ± 0.92	3.50 ± 0.83	0.15	4.60 ± 1.37	4.40 ± 0.92	4.02 ± 1.20	0.24
LDL‐C, mmol/L	2.36 ± 0.74	2.48 ± 0.66	2.11 ± 0.42	0.46	2.84 ± 1.00	2.71 ± 0.80	2.41 ± 0.90	0.29
VLDL‐C, mmol/L	0.59 ± 0.36	0.62 ± 0.32	0.33 ± 0.20	0.25	0.66 ± 0.41	0.62 ± 0.49	0.56 ± 0.21	0.77
HDL‐C, mmol/L	1.11 ± 0.25	1.18 ± 0.25	1.05 ± 0.24	0.28	1.09 ± 0.28	1.09 ± 0.26	1.02 ± 0.30	0.64
ApoAI, g/L	1.12 ± 0.16	1.16 ± 0.16	1.08 ± 0.17	0.39	1.13 ± 0.17	1.11 ± 0.20	1.13 ± 0.17	0.88
ApoB, g/L	0.72 ± 0.23	0.76 ± 0.21	0.59 ± 0.12	0.26	0.83 ± 0.25	0.83 ± 0.25	0.67 ± 0.16	0.10
Lp(a), mg/L	180.19 ± 141.18	230.78 ± 261.41	301.82 ± 344.54	0.43	339.76 ± 377.15	321.08 ± 339.10	423.06 ± 469.33	0.67
UA, μmol/L	325.00 ± 123.51	304.14 ± 97.65	263.60 ± 63.10	0.50	341.38 ± 127.66	317.21 ± 93.78	305.15 ± 101.90	0.35
FPG, mmol/L	5.75 ± 1.41	5.81 ± 2.09	5.09 ± 0.58	0.80	6.30 ± 2.72	6.73 ± 2.98	8.53 ± 5.81^a^	0.03
CysC, mg/L	0.73 ± 0.20	0.71 ± 0.16	0.77 ± 0.35	0.75	0.77 ± 0.25	0.77 ± 0.29	0.75 ± 0.15	0.96
hs‐CRP, mg/L	4.39 ± 6.77	2.34 ± 2.42	2.28 ± 1.26	0.10	7.83 ± 18.24	8.19 ± 17.74	3.65 ± 3.96	0.71
Homocysteine, μmol/L	11.85 ± 3.21	12.49 ± 4.80	14.42 ± 3.02	0.47	12.96 ± 3.00	12.37 ± 3.15	16.80 ± 4.29^a^, ^b^	0.27

Abbreviation: ApoAI, apolipoprotein AI; ApoB, apolipoprotein B; BMI, body mass index; CAD, coronary artery disease; CysC, cystatin c; DBP, diastolic blood pressure; FPG, fasting plasma glucose; HDL‐C, high‐density lipoprotein cholesterol; HR, heart rate; hs‐CRP, hypersensitive C reactive protein; LDL‐C, low‐density lipoprotein cholesterol; Lp(a), lipoprotein (a); MTHFR, 5,10‐methylenetetrahydrofolate reductase; SBP, systolic blood pressure; TC, total cholesterol; TG, triglycerides; UA, Uric acid; VLDL‐C, very low‐density lipoprotein cholesterol.

^a^ *p* < 0.05 compared with that of the CC genotype.

^b^ *p* < 0.05 compared with that of the CT genotype.

### 3.4 Impacts of rs1801131 on CAD susceptibility and severity of coronary lesions

**FIGURE 2:**
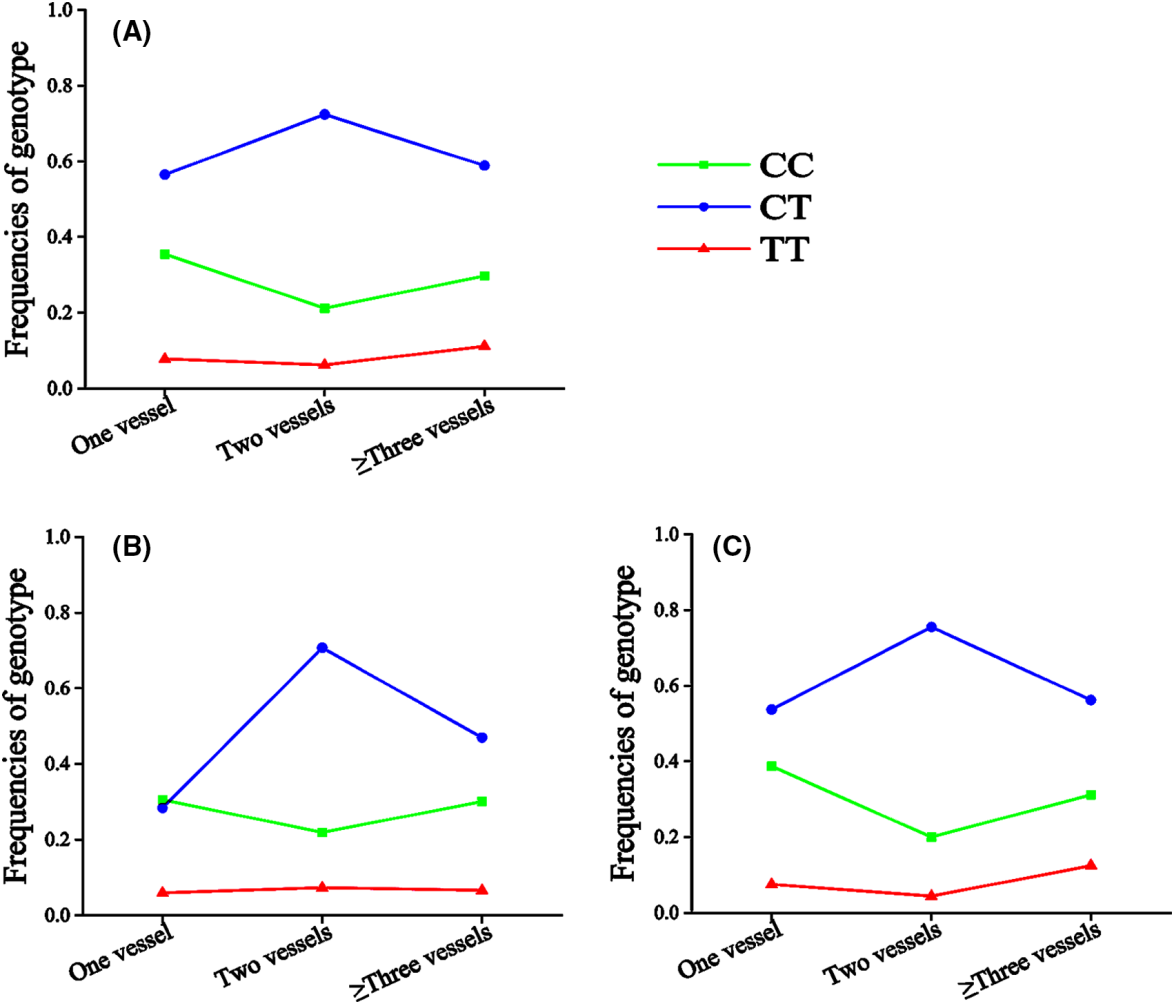
The frequencies of genotypes among the patients with different number of stenotic coronary arteries. (A) All patients with CAD. (B) Male patients with CAD. (C) Female patients with CAD.

**FIGURE 3:**
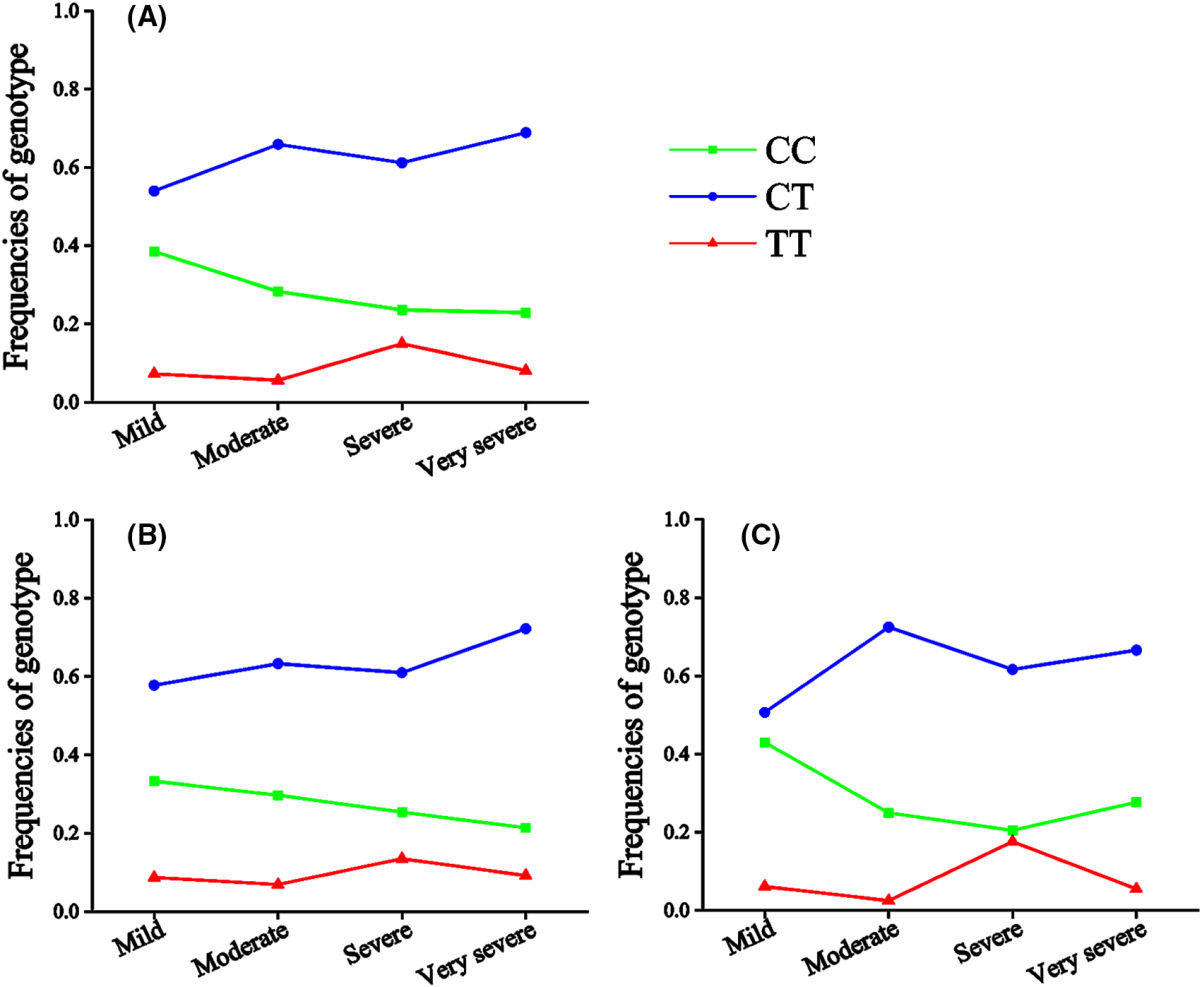
The frequencies of genotypes among the patients with different extent of coronary stenosis. (A) All patients with CAD. (B) Male patients with CAD. (C) Female patients with CAD.

The genotype (CAD group: CC vs. CT vs. TT = 0.29 vs. 0.62 vs. 0.09; Control group: CC vs. CT vs. TT = 0.41 vs. 0.54 vs. 0.05, *p* = 0.01) and allele (CAD group: C vs. T = 0.60 vs. 0.40; Control group: C vs. T = 0.68 vs. 0.32, *p* = 0.01) frequencies of rs1801131 were significantly different between patients with CAD and control individuals (Table 1), suggesting that the T allele of rs1801131 significantly increased the risk of CAD. In addition, the genotype frequencies of rs1801131 were significantly different between patients with different numbers of stenotic coronary arteries (one vessel: CC vs. CT vs. TT = 0.355 vs. 0.565 vs. 0.078; Two vessels: CC vs. CT vs. TT = 0.212 vs. 0.724 vs. 0.062, ≥Three vessels: CC vs. CT vs. TT = 0.298 vs. 0.589 vs. 0.112, *p* = 0.04) (Table 3, Figure 2) or extent of coronary stenosis (Mild: CC vs. CT vs. TT = 0.385 vs. 0.54 vs. 0.073; Moderate: CC vs. CT vs. TT = 0.283 vs. 0.659 vs. 0.056, Severe: CC vs. CT vs. TT = 0.236 vs. 0.612 vs. 0.15, Very severe: CC vs. CT vs. TT = 0.229 vs. 0.689 vs. 0.081, *p* = 0.03) (Table 4, Figure 3), suggesting that rs1801131 was significantly associated with the severity of coronary lesions. Further analyses revealed that the rs1801131 T allele increased the risk of CAD in male individuals (*p* < 0.01) (Table 1), whilst the rs1801131 TT genotype increased the extent of coronary stenosis in female patients with CAD (*p* = 0.04) (Table 4, Figure 3).

**TABLE 3:** The frequencies of genotypes or alleles among the patients with different number of stenotic coronary arteries.

	One vessel	Two vessels	≥Three vessels	*p* values
*MTHFR* rs1801133 (All individuals)				
CC, *n* (%)	54 (35.5)	27 (21.2)	45 (29.8)	0.04
CT, *n* (%)	86 (56.5)	92 (72.4)	89 (58.9)	
TT, *n* (%)	12 (7.8)	8 (6.2)	17 (11.2)	
C allele, *n* (%)	194 (63.8)	146 (57.4)	179 (59.2)	0.28
T allele, *n* (%)	110 (36.1)	108 (42.5)	123 (48.4)	
*MTHFR* rs1801133 (Male individuals)				
CC, *n* (%)	23 (30.6)	18 (21.9)	35 (30.1)	0.45
CT, *n* (%)	43 (28.4)	58 (70.7)	71 (47.0)	
TT, *n* (%)	9 (5.9)	6 (7.3)	10 (6.6)	
C allele, *n* (%)	89 (59.3)	94 (57.3)	141 (60.7)	0.78
T allele, *n* (%)	61 (40.6)	70 (42.6)	91 (55.4)	
*MTHFR* rs1801133 (Female individuals)				
CC, *n* (%)	31 (38.7)	9 (20.0)	10 (31.2)	0.13
CT, *n* (%)	43 (53.7)	34 (75.5)	18 (56.2)	
TT, *n* (%)	6 (7.5)	2 (4.4)	4 (12.5)	
C allele, *n* (%)	105 (65.6)	52 (57.7)	38 (59.3)	0.41
T allele, n (%)	55 (34.3)	38 (42.2)	26 (40.6)	

Abbreviation: MTHFR, 5,10‐methylenetetrahydrofolate reductase.

**TABLE 4:** The frequencies of genotypes or alleles among the patients with different extent of coronary stenosis.

	Mild	Moderate	Severe	Very severe	*p* values
*MTHFR* rs1801133 (All individuals)					
CC, *n* (%)	47 (38.5)	40 (28.3)	22 (23.6)	17 (22.9)	0.03
CT, *n* (%)	66 (54.0)	93 (65.9)	57 (61.2)	51 (68.9)	
TT, *n* (%)	9 (7.3)	8 (5.6)	14 (15.0)	6 (8.1)	
C allele, *n* (%)	160 (65.5)	173 (61.3)	101 (54.3)	85 (57.4)	0.09
T allele, *n* (%)	84 (34.4)	109 (38.6)	85 (45.6)	63 (42.5)	
*MTHFR* rs1801133 (Male individuals)					
CC, *n* (%)	19 (33.3)	30 (29.7)	15 (25.4)	12 (21.4)	0.65
CT, *n* (%)	33 (57.8)	64 (63.3)	36 (61.0)	39 (72.2)	
TT, *n* (%)	5 (8.7)	7 (6.9)	8 (13.5)	5 (9.2)	
C allele, *n* (%)	71 (62.2)	124 (61.3)	66 (55.9)	63 (56.2)	0.62
T allele, *n* (%)	43 (37.7)	78 (38.6)	52 (44.0)	49 (43.7)	
*MTHFR* rs1801133 (Female individuals)					
CC, *n* (%)	28 (43.0)	10 (25.0)	7 (20.5)	5 (27.7)	0.04
CT, *n* (%)	33 (50.7)	29 (72.5)	21 (61.7)	12 (66.6)	
TT, *n* (%)	4 (6.1)	1 (2.5)	6 (17.6)	1 (5.5)	
C allele, *n* (%)	89 (68.4)	49 (61.2)	35 (51.4)	22 (61.6)	0.13
T allele, *n* (%)	41 (31.5)	31 (38.7)	33 (48.5)	14 (38.8)	

Abbreviations: MTHFR, 5,10‐methylenetetrahydrofolate reductase.

### 3.5 Multivariate logistic regression

Multivariate logistic regression was performed to identify independent risk factors for CAD. As shown in Table S2, rs1801133 (*p* < 0.01), age (*p* < 0.01), smoking (*p* = 0.02), weight (*p* = 0.03), BMI (*p* = 0.04), Lp(a) (*p* = 0.01) and hs‐CRP (*p* = 0.03) showed statistically significant in the multivariate logistic regression model. It indicated that these parameters were independently associated with CAD.

### 3.6 ROC curve and PR curve

**FIGURE 4:**
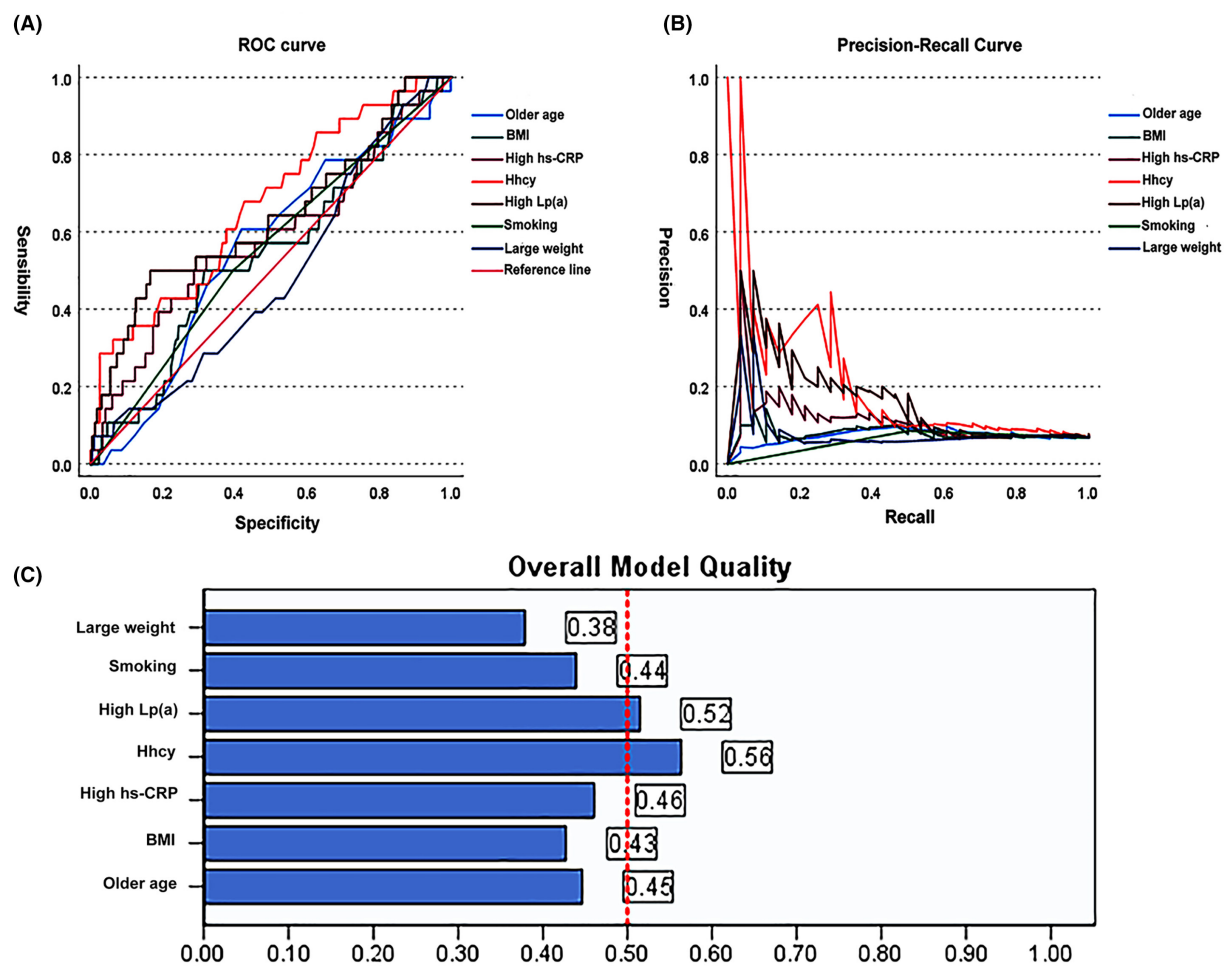
Receiver operating characteristic curve and precision‐recall curve identify key risk factor for severe coronary artery disease. (A) Receiver operating characteristic curve analysis. (B) Precision‐recall curve analysis. (C) Overall model quality. BMI, body mass index; hs‐CRP, hypersensitive C reactive protein; Hhcy, hyperhomocysteinemia; Lp(A), lipoprotein (A).

To investigate whether hyperhomocysteinemia is a determinant of the severity of coronary lesions in the Chinese population. receiver operating characteristic (ROC) curve and precision‐recall (PR) curve analyses were performed, as shown in Figure 4A–C, both ROC (*p* < 0.001) (Figure 4A) and PR curves (*p* < 0.001) (Figure 4B) indicated that hyperhomocysteinemia had good performance in predicting the severity of coronary lesions, whose area under the curve (AUC) (0.669) was larger than other risk factors, including high Lp(a) levels (0.636), high hs‐CRP levels (0.582), older age (0.553), smoking (0.551), high BMI value (0.540) and large body weight (0.485). It indicated that hyperhomocysteinemia was sensitive and accurate for the prediction of the severity of CAD in the Chinese Han population (Figure 4).

## 4 DISCUSSION

The T allele of rs1801133 significantly increased plasma homocysteine levels (Table 2), since rs1801133 was linked to the risk of CAD (Table 1) and severity of coronary lesions (Table 3, Table 4), it indicated that the impacts of rs1801133 on the risk (Table 1) and severity of CAD (Table 3, Table 4) were possibly mediated by high homocysteine levels or hyperhomocysteinemia (Table 2). Mechanistically, hyperhomocysteinemia may promote the occurrence and progress of CAD by inducing inflammatory reaction, fibrosis, oxidative stress, cell proliferation, endothelial dysfunction, thrombosis, atherosclerosis and homocysteinylation of blood proteins of coagulation.^26^, ^33^, ^34^

In male patients with CAD, the carriers of the CT genotype had higher homocysteine, SBP, LDL‐C and hs‐CRP levels and lower APOA1 levels than those with the CC genotype (Table 2). Since cardiometabolic disorder^12^, ^13^, ^14^, ^15^ was related to the risk of CAD, it indicated that the impact of rs1801133 on CAD in male patients (Table 1) was mediated by the impacts of rs1801133 on homocysteine, SBP, LDL‐C, hs‐CRP and APOA1 (Table 2). Three plausible mechanisms could be proposed to explain why rs1801133 affects cardiometabolic parameters. (1) The function of MTHFR polypeptide has been influenced by the amino acid residue substitution (Ala by Val). Different amino acids have different properties, so the molecular function of the MTHFR polypeptide may have been modified if the amino acid residue is replaced by another one. (2) rs1801133 may influence cardiometabolic parameters by modulating the methylation state of DNA or proteins. 5‐MTHF is not only the methyl donor for homocysteine but for many other target molecules, including DNA and proteins.^35^ Conceivably, the functions of the genes or proteins involved in cardiometabolic will be affected if their methylation state changes. (3) Hyperhomocysteinemia is closely associated with the cardiometabolic disorder (e.g. higher LDL‐C, SBP and lower APOA1),^9^, ^10^, ^11^ which indicates that the impacts of rs1801133 on SBP, LDL‐C, APOA1 and hs‐CRP levels in male patients with CAD are possibly mediated by hyperhomocysteinemia.

In female patients with CAD, the TT genotype of rs1801133 significantly increased the extent of coronary stenosis (Table 4). It indicated that female patients with rs1801133 TT genotype were at high risk of severe coronary stenosis. In addition, since the T allele of rs1801133 significantly increased homocysteine levels (Table 2), it indicated that the increased extent of coronary stenosis in female patients (Table 4) was mediated by high homocysteine levels or hyperhomocysteinemia (Table 2). Nevertheless, the impact of rs1801133 on CAD in female individuals did not show statistically significant (Table 1). Two possible reasons could be used to explain this phenomenon. (1) The distribution frequency of the rs1801133 T allele was almost no difference in female patients with CAD (37.8%) and female individuals without CAD (34.1%). (2) Only 255 female individuals were included, which might reduce the statistical power and even cause type II error (false‐negative results). Therefore, future large‐scale clinical trials in Chinese female individuals are certainly needed. In turn, since the sample size of male individuals was relatively larger than female individuals (392 vs. 255), and there was a significant difference in the distribution frequency of rs1801133 T between male patients with CAD (40.6%) and male individuals without CAD (30.2%), it is reasonable to observe that the impact of rs1801133 on CAD risk was significant in male individuals.

According to the 2018 American College of Cardiology (ACC)/American Heart Association (AHA),^36^ the 2019 European Society of Cardiology (ESC)/European Atherosclerosis Society (EAS),^37^ and the Adult Treatment Panel III (ATP III) cholesterol guidelines,^38^ LDL‐C was considered the major cause of CAD and treated as the primary target for therapy, while other lipids were used as the secondary or supplementary therapeutic targets. In this study, the carriers of the TT genotype had higher LDL‐C levels than those carriers of the CC genotype, indicating that healthy male individuals with rs1801133 TT genotype were at high risk of CAD.

The TT genotype of rs1801133 was found to increase homocysteine levels in CAD and CAD‐free individuals (Table 2), consistent with Bickel et al. finding.^39^ Since individuals with TT genotype were predisposed to mild to moderate hyperhomocysteinemia,^6^, ^7^, ^8^ and hyperhomocysteinemia was closely associated with the risk of CAD^22^ and severity of CAD,^40^ it indicated that those individuals (i.e. CAD‐free individuals and female patients with CAD) with TT genotype (Table 2) were at high risk of CAD or severe CAD. Currently, dietary supplementation of folic acid and B vitamins is widely used in clinical practice for the primary and secondary prevention of CAD via down‐regulating plasma homocysteine levels or inhibiting hyperhomocysteinemia,^41^, ^42^, ^43^, ^44^, ^45^, ^46^ and this fortification measure is proven to be safe and effective.^41^, ^42^, ^43^, ^44^, ^45^, ^46^ Therefore, for those high‐risk individuals (i.e. CAD‐free individuals, male patients with CAD, and female patients with CAD) with high homocysteine levels or hyperhomocysteinemia (Table 2), dietary vitamin supplementation (i.e. folic acid, B6, and B12) should be recommended for the primary and secondary prevention of CAD. However, RCTs are needed to verify this hypothesis and detail the dose, frequency, and duration of supplementation.

The present logistic regression indicated that older age, smoking, large body weight, high BMI value, high Lp(a) levels and high hs‐CRP levels were independent risk factors for CAD (Table S2), consistent with previous findings.^47^, ^48^, ^49^, ^50^, ^51^, ^52^ Despite Zhu et al.^53^ found that rs1801133 was an independent risk factor for clinical outcomes in CAD subjects treated with statins. This study directly demonstrated that the rs1801133 variant was an independent risk factor for CAD (Table S2). More importantly, ROC curve and PR curve analyses indicated that hyperhomocysteinemia was sensitive and accurate for the prediction of the severity of CAD in the Chinese Han population (Figure 4).

Our previous study^54^ indicated that the T allele of rs1801133 was strongly associated with CAD in Asians, in line with findings from this study whereby the T allele of rs1801133 significantly increased the risk of CAD and severity of coronary lesions in the Chinese Han population. Mechanistically, our previous study^54^ reckoned that the relationship between rs1801133 and CAD was possibly and partly mediated by abnormal lipid levels (i.e. high LDL‐C and TC levels). In contrast, this study revealed that the impacts of rs1801133 on CAD (Table 1) and severity of coronary lesions (Table 3, Table 4) were primarily mediated by high homocysteine levels or hyperhomocysteinemia (Table 2), but not dyslipidaemia (Table 2). Two possible reasons could be utilized to explain these inconsistencies or contradictions. (1) Our previous study^54^ did not include plasma homocysteine levels for the analysis, therefore, we did not have a chance to detect the impact of rs1801133 on plasma homocysteine levels. (2) Our previous study^54^ included 85,554 individuals for the lipid association analysis, the statistical power was adequate to detect the real impacts of rs1801133 on plasma lipid levels. In contrast, this study only included 646 Chinese adult individuals for lipid association analysis (Table 2), which might reduce the statistical power and thus manifested negative results in the lipid association analysis. It in turn indicated that the impacts of rs1801133 on CAD and severity of coronary lesions were more possibly and primarily mediated by hyperhomocysteinemia, followed by dyslipidaemia.

The present study has several limitations. First, the participants in the control group were those who underwent angiography with suspected CAD at our hospital and were not healthy individuals. It may lead to a selection bias, but it is difficult to enrol healthy subjects from general population who are willing to undergo coronary angiography in this kind of study. Second, the sample size of the control group is relatively small and this may limit the statistical power in the analyses. Third, all the participants enrolled in this study were Chinese Han people and therefore the findings from this study may not apply to other ethnic origins.

## 5 CONCLUSIONS

The increased risk of CAD and severity of coronary lesions associated with rs1801133 in the Chinese Han population were attributed, at least partly, to high homocysteine levels. Hyperhomocysteinemia had a high predictive value for severe CAD or multiple vessel lesions.

## AUTHOR CONTRIBUTIONS

**Zhi Luo:** Conceptualization (lead); supervision (equal); writing – original draft (lead); writing – review and editing (lead). **Kai Tang:** Data curation (equal); investigation (equal). **Gang Huang:** Data curation (equal); investigation (equal). **Xiao Wang:** Data curation (equal); investigation (equal). **Shiheng Zhou:** Data curation (equal); investigation (equal). **Daying Dai:** Data curation (equal); investigation (equal). **Hanxuan Yang:** Data curation (equal); investigation (equal). **Wencai Jiang:** Data curation (equal); investigation (equal).

## FUNDING INFORMATION

This study did not receive any funding in any form.

## CONFLICT OF INTEREST STATEMENT

The authors confirm that there are no conflicts of interest.

## Supporting information


Table S1.

## Data Availability

The data that support the findings of this study are available from the corresponding author upon reasonable request.
