# Supplementary material for: Homocysteine concentration in coronary artery disease and severity of coronary lesions
Source: J Cell Mol Med. 2024 Jun 19;28(12):e18474. doi: 10.1111/jcmm.18474 (PMC11187881; doi:10.1111/jcmm.18474)
Supplement: Supplementary file 1 — Table S1. [file JCMM-28-e18474-s001.docx]

*Table S1* **Clinical characteristics of the study population.**

| **Cardiometabolic variables** | **Control group (n=216)** | **CAD group (n=430)** | ***P* values** |
| --- | --- | --- | --- |
| Age, years | 61.2±10.06 | 64.6±9.30 | <0.001 |
| Height, cm | 161.91±7.25 | 161.69±7.49 | 0.73 |
| Weight, kg | 63.41±9.99 | 62.38±9.67 | 0.21 |
| BMI, kg/m^2^ | 24.20±3.28 | 23.86±3.24 | 0.23 |
| HR, beats/minute | 74.28±14.42 | 76.16±14.54 | 0.12 |
| SBP, mmHg | 136.49±24.32 | 145.41±27.87 | <0.001 |
| DBP, mmHg | 83.82±14.63 | 89.33±16.12 | <0.001 |
| TG, mmol/L | 1.43±0.82 | 1.62±1.27 | 0.05 |
| TC, mmol/L | 4.06±0.97 | 4.20±1.18 | 0.12 |
| LDL-C, mmol/L | 2.41±0.70 | 2.60±0.94 | 0.01 |
| VLDL-C, mmol/L | 0.56±0.33 | 0.59±0.41 | 0.44 |
| HDL-C, mmol/L | 1.10±0.30 | 1.01±0.26 | <0.001 |
| ApoAI, g/L | 1.13±0.18 | 1.05±0.20 | <0.001 |
| ApoB, g/L | 0.74±0.23 | 0.80±0.27 | 0.01 |
| Lp(a), mg/L | 210.00±225.23 | 317.53±344.52 | <0.001 |
| UA, μmol/L | 347.21±111.49 | 361.73±116.31 | 0.13 |
| FPG, mmol/L | 5.93±2.47 | 6.68±3.17 | <0.01 |
| CysC, mg/L | 0.75±0.21 | 0.84±0.51 | 0.03 |
| hs-CRP, mg/L | 4.60±9.89 | 10.62±21.77 | <0.001 |
| Homocysteine, μmol/L | 13.78±4.95 | 18.23±4.86 | <0.001 |

CAD: coronary artery disease; BMI: body mass index; HR: heart rate; SBP: systolic blood pressure; DBP: diastolic blood pressure;

TG: triglycerides; TC: total cholesterol; LDL-C: low-density lipoprotein cholesterol; VLDL-C: very low-density lipoprotein

cholesterol; HDL-C: high-density lipoprotein cholesterol; ApoAI: apolipoprotein AI ;ApoB: apolipoprotein B; Lp(a): lipoprotein (a);

UA: uric acid; FPG: fasting plasma glucose; CysC: cystatin c; hs-CRP: hypersensitive C reactive protein.

*Table S2* **Multivariate logistic regression analysis.**

| Covariates | Exp(B) | 95% CI | *P* values |
| --- | --- | --- | --- |
| rs1801133 | 0.56 | 0.38-0.82 | <0.01 |
| Age | 0.96 | 0.94-0.98 | <0.01 |
| Height | 0.83 | 0.66-1.03 | 0.09 |
| Weight | 1.37 | 1.03-1.83 | 0.03 |
| BMI | 0.47 | 0.23-0.98 | 0.04 |
| HR | 0.99 | 0.97-1.01 | 0.35 |
| Smoking | 0.52 | 0.29-0.91 | 0.02 |
| Hypertension | 0.86 | 0.66-1.12 | 0.26 |
| Diabetes | 0.56 | 0.27-1.14 | 0.11 |
| SBP | 1.00 | 0.98-1.02 | 0.68 |
| DBP | 0.98 | 0.56-1.00 | 0.08 |
| TG | 0.86 | 0.59-1.26 | 0.46 |
| TC | 0.03 | 0.00-7.08 | 0.21 |
| LDL-C | 15.78 | 0.07-3.29 | 0.31 |
| VLDL-C | 37.41 | 0.16-8.39 | 0.19 |
| HDL-C | 5.89 | 0.21-16.35 | 0.15 |
| ApoAI | 3.19 | 0.31-3.23 | 0.32 |
| ApoB | 2.38 | 0.16-3.56 | 0.53 |
| Lp(a) | 0.99 | 0.99-1.00 | 0.01 |
| UA | 1.00 | 0.99-1.00 | 0.67 |
| FPG | 0.94 | 0.82-1.08 | 0.41 |
| CysC | 0.83 | 0.34-2.02 | 0.68 |
| hs-CRP | 0.98 | 0.96-0.99 | 0.03 |
| Homocysteine | 1.00 | 0.95-1.06 | 0.77 |

BMI: body mass index; HR: heart rate; SBP: systolic blood pressure; DBP: diastolic blood pressure;TG: triglycerides;

TC: total cholesterol; LDL-C: low-density lipoprotein cholesterol; VLDL-C: very low-density lipoprotein cholesterol;

HDL-C: high-density lipoprotein cholesterol; ApoAI: apolipoprotein AI ;ApoB: apolipoprotein B; Lp(a): lipoprotein (a);

UA: uric acid; FPG: fasting plasma glucose; CysC: cystatin c; hs-CRP: hypersensitive C reactive protein.
